# Supplementary material for: Collaborative intelligence in AI: Evaluating the performance of a council of AIs on the USMLE
Source: PLOS Digit Health. 2025 Oct 9;4(10):e0000787. doi: 10.1371/journal.pdig.0000787 (PMC12510544; doi:10.1371/journal.pdig.0000787)
Supplement: S2 File — (ZIP) [file pdig.0000787.s002.zip › S2 Data Raw data files containing unprocessed question inputs and LLM outputs including transcripts of deliberations/STEP3_Question-132-1_Type-MC-NJ.docx]

**Facilitator** **[Round 1]:** A 72-year-old man is admitted to the intensive care unit for pain management and observation of ventricular ectopy 4 hours after undergoing uncomplicated left total knee replacement. He is receiving fentanyl via systemic patient-controlled analgesic pump for pain management. Medical history is remarkable for coronary artery disease, for which he takes atorvastatin, furosemide, carvedilol, lisinopril, and 81-mg aspirin. Baseline echocardiogram showed an ejection fraction of 0.20. Vital signs now are temperature 38.0°C (100.4°F), pulse 90/min, respirations 16/min, and blood pressure 130/90 mm Hg. Pulse oximetry on room air shows an oxygen saturation of 96%. Physical examination discloses no unexpected abnormalities. Which of the following regimens for prophylaxis of deep venous thrombosis will likely be most beneficial in this patient? (A) Continuous application of bilateral lower extremity pneumatic compression devices (B) Continuous intravenous infusion of heparin titrated to a PTT of 1.5 to 2.0 times the control value (C) Oral warfarin (D) Subcutaneous enoxaparin (E) Subcutaneous heparin

**AI_A:** RESTATING THE QUESTION: The question is essentially asking us which treatment option would be best for this patient for preventing deep vein thrombosis (DVT). DVT is a condition where blood clots form in the deep veins of the body, most commonly the leg, and this clot can potentially break off and cause a life-threatening embolism. The patient has recently gone through surgery and has a history of heart disease, both of which are risk factors for DVT. PLANNING: Several assumptions are being made here. One is that the patient's ventricular ectopy and heart disease could potentially impact which DVT prophylaxis is most safe and effective. We would generally like to use medication to prevent DVT in this postoperative patient; however, his low ejection fraction or the degree of heart's blood pumping function may complicate this. It's also important to consider his other medications, which include drugs that also have an impact on coagulation, like Aspirin. A second assumption is that the patient's ability to use subcutaneous injections or oral medications may be affected by his health state and heart function. CLINICAL REASONING: Given the patient's medical history and current state, it seems reasonable to hypothesize that the safest and most effective prophylaxis against DVT might come from local measures that do not interact with systemic coagulation or heart function directly. The choice would need to balance the benefits of DVT prevention with the risks involved with systemic anticoagulation, especially given his current medication, cardiac status, and recent operative procedure. CONCLUDING: Among the options provided, the continuous application of bilateral lower extremity pneumatic compression devices stands out as the possibly optimal method. This specifically applies gentle pressure to the legs to encourage blood flow and works locally without systemic effects. This would avoid potential complications with systemic anticoagulation medication and the potential for added strain on the heart or alterations in his coagulation status given his current medication regimen. RESPONSE: Therefore, the best DVT prophylaxis in this case would be (A) Continuous application of bilateral lower extremity pneumatic compression devices.

**AI_B:** 1) RESTATING THE QUESTION: The elderly man in question underwent a left total knee replacement and is now in the intensive care unit for pain relief and to monitor radar ectopy. He receives fentanyl for pain and has a recorded medical history of coronary artery disease and is on multiple medications. After the surgery, his vital signs are stable except for a slightly elevated body temperature. The critical question is which regimen out of the options provided would be the most effective prevention for deep vein thrombosis. 2) PLANNING: DVT prophylaxis after orthopedic surgery is crucial. Several approaches exist based on individual patient risk factors. The choices here include mechanical (pneumatic compression devices), and pharmacological options (heparin, enoxaparin, warfarin). This patient's low ejection fraction and comorbidities suggest a heightened risk for complications from more aggressive anticoagulation therapies. Therefore, the suitability of each method in the light of the patient's cardiovascular status and the efficacy of DVT prevention has to be considered. 3) CLINICAL REASONING: Bilateral lower extremity pneumatic compression devices are a non-pharmacological option that may reduce the risk of bleeding compared to anticoagulation therapies. However, their effectiveness is less than that of pharmacological treatments. IV heparin, warfarin, and subcutaneous heparin/enoxaparin are all potential treatments, but they carry a risk of bleeding especially in a patient with low ejection fraction. The choice between these options would depend on the balance between the risk of bleeding and the risk of DVT. In that case, subcutaneous heparin or enoxaparin, which carry less bleeding risk than heparin titrated to a PTT of 1.5-2.0 and warfarin, may be more appropriate. 4) CONCLUDING: Weighing up the bleeding risks and DVT prophylaxis efficacy, subcutaneous medical options appear to be the prudent middle ground. The choice among them would likely be between heparin and enoxaparin. Enoxaparin is a low-molecular-weight heparin (LMWH) which is known to have a more predictable anticoagulant response, less monitoring needs as compared to unfractionated heparin. 5) RESPONSE: Therefore, the most beneficial regimen for prophylaxis of deep venous thrombosis in this patient will likely be subcutaneous enoxaparin (D).

**AI_D:** (1) RESTATING THE QUESTION: The question asks which regimen will be most beneficial in preventing deep venous thrombosis (DVT) in the case of an elderly man who's just undergone left total knee replacement and is currently under pain management and observation related to his heart - specifically ventricular ectopy. The patient has a documented history of coronary artery disease and is on medication including atorvastatin, furosemide, carvedilol, lisinopril, and 81-mg aspirin. His baseline echocardiogram indicated an ejection fraction of 0.20. Current vital observations show mildly elevated body temperature, normal pulse rate, slightly reduced level of respirations and slightly higher than normal blood pressure. (2) PLANNING: The assumptions being made here include that the patient's past and current medical conditions and medications may impact the recommended regimen and that all the proposed regimens are available options. If we question these assumptions, we could hypothesize that the patient's severe heart disease (indicated by low ejection fraction) might make certain clotting-related medications risky. Also, his ongoing pain management might impact the type of regimen that can be used. The primary clinical priority would be to use a regimen that is least likely to exacerbate his heart condition or interfere with his pain management. (3) CLINICAL REASONING: The patient's reported cardiac history and low ejection fraction raise concerns about the safety of high-risk anticoagulants. Potential bleeding and impact on his reduced cardiac output must be considered. Further, he is already receiving fentanyl for pain management. Certain anticoagulants may interact with underlying conditions or medications potentially causing complications. (4) CONCLUDING: Considering the patient's age, comorbidities, and post-operative status from knee surgery, it's important to choose a DVT prophylaxis measure that offers optimal effectiveness while minimizing potential risks. In general, anticoagulation should be used in the postoperative period following total knee replacement - this reduces the risk of DVT. Among the given options, enoxaparin (D) is frequently used due to its efficacy and safety profile. (5) RESPONSE: In this patient, given the considerations discussed, the regimen likely to be most beneficial for deep venous thrombosis prophylaxis is (D) subcutaneous enoxaparin.

**AI_E:** The patient is a 72-year-old man who has just undergone a knee replacement surgery and is now in the intensive care unit. His medical history includes coronary artery disease, for which he is on several medications. With an ejection fraction of 0.20 indicating impaired cardiac function, and being postoperative for a major surgery, he is at an increased risk for deep vein thrombosis (DVT). The proposed interventions are aimed at reducing this risk. DVT is essentially a blood clot that forms within a vein, often due to stagnation of blood or hypercoagulability, and major surgery, especially involving the lower extremities, is a significant risk factor. In addressing this question, we need to consider both the efficacy of the prophylactic interventions and the patient's overall health status. For instance, medications like heparin and warfarin would thin the blood and prevent clot formation, but may pose a risk for this patient with cardiac disease. On the other hand, physical interventions like pneumatic compression devices can also help prevent DVT, but may not be as effective as anticoagulants. Weighing these factors, it seems clear that the best course of action would be a balance of efficacy and safety. Taking into account his history of coronary artery disease, atorvastatin, furosemide, carvedilol, lisinopril and low-dose aspirin are being used. These do not preclude the use of anticoagulants, but caution and proper monitoring should be maintained to avoid potential bleeding complications. Given his recent surgery and the reduced ejection fraction i.e., decreased pumping capacity of his heart, the risk of DVT is elevated, so the use of prophylactic anticoagulation would be considered standard of care. Enoxaparin and heparin are both effective, but enoxaparin has the benefit of more predictable pharmacokinetics and anti-factor Xa activity, making it the preferred choice in many situations. On the other hand, oral warfarin's onset is slow, making it less beneficial in an immediate postoperative setting. Hence, his best option for DVT prophylaxis would likely be subcutaneous enoxaparin. In conclusion, in a postoperative patient with decreased ejection fraction and on multiple cardiac medications, the use of subcutaneous enoxaparin, considering its predictability and efficacy, seems the most beneficial course for DVT prophylaxis. Answer: (D) Subcutaneous enoxaparin.

**AI_C:** (1) RESTATING THE QUESTION: The scenario describes a 72-year-old male patient who has undergone a left total knee replacement. This patient has underlying coronary artery disease, and he is currently in the intensive care unit (ICU) for pain management as well as continuous monitoring of a cardiac rhythm disturbance, namely, ventricular ectopy. He is placed on an analgesic protocol that includes fentanyl administration via a patient-controlled pump. Considering his medical history, and current physiological state, it's important to determine the optimal regimen to prevent a common postoperative complication known as deep vein thrombosis (DVT) - which is the formation of a blood clot within a deep vein typically in the leg. (2) PLANNING: Preserving the health of a patient during the postoperative period is a delicate process that involves balancing several factors. In this scenario, the patient's risk for DVT is high due to his age, immobilization, and the nature of the procedure. However, his pathologic cardiac rhythm (ventricular ectopy) and compromised heart function (ejection fraction of 0.20) limits the use of aggressive anticoagulant regimens due to higher risk for bleeding which can exacerbate his cardiac status. These assumptions question the potential benefits and drawbacks of each of the proposed interventions to determine which would be the most effective in preventing DVT while minimizing the risk of complications given the patient’s state. (3) CLINICAL REASONING: Regarding the proposed regimens, oral warfarin (option C) and Subcutaneous enoxaparin (option D) are potent anticoagulants but they may place the patient at a heightened risk of bleeding. Because of the patient's fragile cardiac health, uncontrolled hemorrhage can lead to catastrophic outcomes. The same applies to continuous IV infusion of heparin (option B) to a PTT of 1.5 to 2.0 times the control value. On the other hand, continuous application of bilateral lower extremity pneumatic compression devices (option A) may not provide sufficient prophylaxis against DVT as it solely depends on encouraging venous return and does not possess any anticoagulant properties. Finally, option E, subcutaneous heparin, provides a balance between anticoagulation and a manageable risk profile for bleeding. It's also used in patients with renal insufficiency and does not require the kind of strict monitoring that warfarin requires, which makes it more suitable for a postoperative setting. (4) CONCLUDING: Taking all these points into consideration, the best choice seems to be a regimen that offers an optimized risk-benefit ratio in terms of preventing DVT and avoiding bleeding complications. Subcutaneous heparin is the routine method of applying a chemical DVT prophylaxis and has a documented safety record in coronary artery disease patients. (5) RESPONSE: Based on the information provided and considering the patient's medical history, the best option for DVT prophylaxis for this patient would be subcutaneous heparin (E).

**Facilitator** **[Round 2]:** Summary: AI_A believes that the best treatment for preventing deep vein thrombosis (DVT) in the patient is the continuous application of bilateral lower extremity pneumatic compression devices. This is due to the patient's medical history and current state, which may complicate the use of medication for DVT prevention. The pneumatic compression devices work locally without systemic effects, avoiding potential complications with systemic anticoagulation medication. AI_B, AI_D, and AI_E all agree that subcutaneous enoxaparin is the most beneficial regimen for DVT prophylaxis in this patient. They argue that this treatment strikes a balance between the risk of bleeding and the risk of DVT, and is more predictable and requires less monitoring than other options. AI_C, on the other hand, suggests that subcutaneous heparin is the best option for DVT prophylaxis. This is because it provides a balance between anticoagulation and a manageable risk profile for bleeding, and does not require the kind of strict monitoring that warfarin requires. Question for Council Members: The main disagreement seems to be between the use of pneumatic compression devices and the use of subcutaneous enoxaparin or heparin. Could you please elaborate on the factors that led you to your conclusion, specifically addressing the efficacy and safety of these treatments in the context of this patient's medical history and current state?. Based on your preceding response, how would you answer the original question: [A 72-year-old man is admitted to the intensive care unit for pain management and observation of ventricular ectopy 4 hours after undergoing uncomplicated left total knee replacement. He is receiving fentanyl via systemic patient-controlled analgesic pump for pain management. Medical history is remarkable for coronary artery disease, for which he takes atorvastatin, furosemide, carvedilol, lisinopril, and 81-mg aspirin. Baseline echocardiogram showed an ejection fraction of 0.20. Vital signs now are temperature 38.0°C (100.4°F), pulse 90/min, respirations 16/min, and blood pressure 130/90 mm Hg. Pulse oximetry on room air shows an oxygen saturation of 96%. Physical examination discloses no unexpected abnormalities. Which of the following regimens for prophylaxis of deep venous thrombosis will likely be most beneficial in this patient? (A) Continuous application of bilateral lower extremity pneumatic compression devices (B) Continuous intravenous infusion of heparin titrated to a PTT of 1.5 to 2.0 times the control value (C) Oral warfarin (D) Subcutaneous enoxaparin (E) Subcutaneous heparin]

**AI_A:** To reframe the question, it aims to identify the most effective strategy in the prevention of deep venous thrombosis (DVT) in a 72-year-old postoperative patient with significant cardiovascular co-morbidities, in the context of certain treatment options: pneumatic compression devices, continuous intravenous (IV) heparin, oral warfarin, subcutaneous (SC) enoxaparin, and SC heparin. Planning involves identifying factors that could influence the choice of prophylaxis: patient's age, surgical procedure, cardiovascular history, current medications, and postoperative status. And weighing the benefits and risks of thrombolytic therapies, given patient's ejection fraction of 0.20, and the risk of bleeding and risk for thromboembolic events. By clinically reasoning through these factors - the use of fentanyl for pain management reduces the risk of pulmonary embolism following surgery by influencing venous stasis (a key component of Virchow's triad), but it does not negate the risk of DVT. The patient's coronary artery disease, limited cardiac function (ejection fraction of 0.20), and medication profile significantly elevate his risk of bleeding, making full-dose thrombolytic therapy risky. The patient's post-knee replacement status increases his risk of DVT. In conclusion, despite benefiting from medication for DVT prophylaxis, drugs that alter coagulation might increase the patient's chances of bleeding considering his comorbidities and current medications. Leveraging mechanical thromboprophylaxis options like pneumatic compression devices can reduce the risk of DVT without significantly increasing the risk of bleeding. Given these factors, the best approach for DVT prophylaxis in this patient would be the (A) continuous application of bilateral lower extremity pneumatic compression devices. It provides the beneficial effect of prophylaxis against DVT while avoiding potential systemic complications that come with anticoagulation therapy in a patient with a compromised cardiovascular status.

**AI_B:** Restating the Question: The question aims to identify the most beneficial preventive measure for deep venous thrombosis (DVT) in a 72-year-old male patient who recently underwent a left total knee replacement. This patient's notable medical history includes coronary artery disease (with an ejection fraction of 0.20), and he is taking several cardiac medications. Following surgery, he is being monitored for ventricular ectopy and is receiving fentanyl for pain management. Planning: In planning our answer, we need to consider the characteristics of this patient carefully: older age, recent surgery, and remarkable cardiac history. These factors all increase his risk for DVT. Therefore, DVT prophylaxis is crucial in this patient. The question at hand is which method of prophylaxis is most beneficial, given his specific circumstances. Our clinical decision must balance the effectiveness of DVT prevention, the risk of bleeding presented by certain options, and stability of the patient’s cardiac condition. Clinical Reasoning: All options offered for the prophylaxis of DVT in this patient have their advantages and disadvantages. Pneumatic compression devices (Option A) are non-pharmacological and thus do not impart added risk of bleeding or interact with his current medications. However, their effectiveness compared to pharmacological options is generally less predictable. Intravenous infusion of heparin (Option B) and oral warfarin (Option C) require regular monitoring and dose adjustments, potentially challenging considering the patient's unstable cardiac status and the associated risk of bleeding. Subcutaneous enoxaparin (Option D) and heparin (Option E) are generally less intensive to monitor and carry a lower risk of bleeding complications compared to intravenous heparin and oral warfarin. Concluding: Considering the patient's significant cardiac history, including low ejection fraction, and the balance between DVT prophylaxis and the associated risk of bleeding, using a method with simpler monitoring requirements and less systemic effects would be preferable. Therefore, the injection of a low molecular weight heparin such as enoxaparin, which is subcutaneous, requires less monitoring, and carries a lower risk of bleeding, seems the most beneficial. Response: The prophylaxis of deep venous thrombosis in this patient would likely be most beneficial with subcutaneous enoxaparin (D).

**AI_D:** Restating the question, the inquiry is about which DVT prophylaxis measure is the most beneficial for a 72-year old patient with notable medical history, after a knee replacement surgery. The options present include mechanical and pharmacological interventions such as pneumatic compression devices, intravenous heparin infusion, oral warfarin, and subcutaneous administration of enoxaparin or heparin. The planning phase involves considering the patient's specific details: the patient has low ejection fraction suggesting heart failure, is on medications for coronary artery disease and is currently in pain management post surgery. The assumption here is that the patient's underlying health conditions, particularly his cardiac history, may interact with anticoagulant prophylaxis, hence potentially influencing the choice of DVT prophylaxis. In the clinical reasoning phase, it's important to weigh the benefits and risks of each prophylaxis option. Our primary aim is to prevent DVT, a common potential complication post orthopedic surgeries like knee replacements. However, in achieving this, care must be taken not to worsen the patient's existing health status. With the patient's low ejection fraction, options that carry a high bleeding risk, such as IV heparin infusion or warfarin, could potentially worsen his condition. The use of systemic anticoagulation can be a challenge in patients with low cardiac output. In conclusion, the decision should be a balance between the effectiveness of DVT prophylaxis and the potential risk, particularly in a patient with a critical cardiac profile. Enoxaparin and heparin are both anticoagulants. Enoxaparin, a low molecular weight heparin (LMWH), is preferred because it has predictable anticoagulant response, requires no monitoring and has fewer side effects including bleeding complications compared to unfractionated heparin. For the given patient, my response would, therefore, be (D) Subcutaneous enoxaparin. It provides the needed DVT prophylaxis without putting the patient at unnecessary additional risk by considering his overall clinical context. Pneumatic compression devices may be considered, especially due to this patient's heart condition, however, in effectiveness, pharmacological prophylaxis has the upper hand, and as long as its administration is careful, enoxaparin should be effective and safe.

**AI_E:** 1) RESTATING THE QUESTION: To restate the question in simpler terms, we are asked to decide the best way to prevent deep venous thrombosis (DVT)- a condition where blood clots form in deep veins, often in the leg, in a patient who is 72 years old and has just had a total knee replacement. His current medical condition and history need also to be considered. The patient has coronary artery disease and is currently being managed with several drugs. He also has a low ejection fraction (amount of blood pumped out of the heart), and his vital signs are stable at the moment. 2) PLANNING: We can start by assuming that the patient, due to the nature of the medical procedure performed on him, is at an elevated risk of DVT. The drugs he is currently on, notably atorvastatin (a statin), furosemide (diuretic), carvedilol (beta-blocker), lisinopril (ACE inhibitor), and aspirin (antiplatelet), for the management of his coronary artery disease, can interact with any new medication introduced, either enhancing or reducing the effect of the other. Let's focus on the most clinically significant one — the interaction between antiplatelets/anticoagulants and some of the proposed regimens. 3) CLINICAL REASONING: Because of the invasive procedure performed on the patient, he is at high risk of clotting, leading to DVT, a potentially fatal condition if not appropriately managed. However, the patient's low ejection fraction means that any regimen that exacerbates this condition, such as warfarin, might not be best for this patient. Therefore, either pneumatic devices or subcutaneous heparin/enoxaparin might be the best options for him, depending on the safety and efficacy of both methods. 4) CONCLUDING: After considering the situation, the decision comes down to efficacy and safety. Subcutaneous enoxaparin requires less monitoring and has a predictable anticoagulative effect. In contrast, pneumatic compression devices are safe but perhaps not as effective as drug prevention options. Given the patient's advanced age and precarious health situation, he probably won't tolerate bleeding complications, which makes me lean towards the pneumatic compression devices even more. 5) RESPONSE: Therefore, my answer to your question would be: (A) Continuous application of bilateral lower extremity pneumatic compression devices could be most beneficial as it presents the least risk to the patient, despite possibly lesser efficacy compared to subcutaneous heparin or enoxaparin.

**AI_C:** The case presents a 72-year-old man who recently underwent a total knee replacement and is currently in intensive care for ventricular ectopy and pain control. His medical history includes coronary artery disease, for which he is on multiple medications. He is also receiving fentanyl for pain relief. The goal of prophylaxis in this context is to prevent deep venous thrombosis (DVT), a clotting disorder characterized by the formation of blood clots in the deep veins, typically of the legs. It is crucial to choose the best prophylactic regimen that balances the prevention of DVT while taking into account his cardiovascular risks and bleeding potential. The proposed options include the application of lower extremity pneumatic compression devices and a range of anticoagulant drugs administered via various routes, including continuous intravenous heparin, oral warfarin, and subcutaneous enoxaparin or heparin. We need to consider the individual risks and benefits of these strategies in relation to the patient's overall clinical context. Pneumatic compression devices promote blood flow locally thereby reducing the risk of DVT, but do not have systemic anticoagulant effects that might potentiate his cardiovascular and bleeding risks. Systemic anticoagulants, on the other hand, have an increased risk of systemic complications, which may be risky given the patient's cardiac profile, but offer more reliable prophylaxis against DVT. Between the systemic anticoagulant options, enoxaparin and heparin have some advantages over warfarin and intravenous heparin in this setting. Warfarin has a delayed onset of action and requires careful monitoring of INR, making it less practical in the acute setting. Enoxaparin and heparin are faster acting and have a predictable anticoagulant effect, with enoxaparin requiring less regular blood tests than heparin. In terms of bleeding risk, while all these agents can increase it, the risk could be managed cautiously with heparin due to its short half-life, and antidote availability. Therefore, pivoting from the understanding above, the advisable choice would be the approach which provides effective prophylaxis with manageable bleeding risk, avoids strict monitoring schedule - making subcutaneous heparin a potential choice. Given the above said reasoning and understanding, the most beneficial regimen for prophylaxis of deep venous thrombosis in this patient would likely be Subcutaneous heparin (Option E).

**Facilitator** **[Round 3]:** Summary: AI_A believes that the best approach for DVT prophylaxis in the patient would be the continuous application of bilateral lower extremity pneumatic compression devices. This approach provides the beneficial effect of prophylaxis against DVT while avoiding potential systemic complications that come with anticoagulation therapy in a patient with a compromised cardiovascular status. AI_B suggests that the most beneficial preventive measure for DVT in the patient would be the injection of a low molecular weight heparin such as enoxaparin. This method requires less monitoring, carries a lower risk of bleeding, and does not have systemic effects. AI_C recommends subcutaneous heparin as the most beneficial regimen for prophylaxis of deep venous thrombosis in the patient. This approach provides effective prophylaxis with manageable bleeding risk and avoids a strict monitoring schedule. AI_D suggests that subcutaneous enoxaparin would be the most beneficial for the patient. It provides the needed DVT prophylaxis without putting the patient at unnecessary additional risk by considering his overall clinical context. AI_E believes that the continuous application of bilateral lower extremity pneumatic compression devices could be most beneficial as it presents the least risk to the patient, despite possibly lesser efficacy compared to subcutaneous heparin or enoxaparin. Key Differences: The key differences in the council members' perspectives revolve around the balance between the effectiveness of DVT prophylaxis and the potential risk, particularly in a patient with a critical cardiac profile. AI_A and AI_E lean towards mechanical prophylaxis (pneumatic compression devices) due to its safety, while AI_B, AI_C, and AI_D favor pharmacological prophylaxis (subcutaneous enoxaparin or heparin) due to its effectiveness. Question to Clarify Disagreements: Given the patient's significant cardiovascular comorbidities and the risk of bleeding, how do you weigh the safety of mechanical prophylaxis against the effectiveness of pharmacological prophylaxis? Can you provide more evidence or reasoning to support your chosen approach?. Based on your preceding response, how would you answer the original question: [A 72-year-old man is admitted to the intensive care unit for pain management and observation of ventricular ectopy 4 hours after undergoing uncomplicated left total knee replacement. He is receiving fentanyl via systemic patient-controlled analgesic pump for pain management. Medical history is remarkable for coronary artery disease, for which he takes atorvastatin, furosemide, carvedilol, lisinopril, and 81-mg aspirin. Baseline echocardiogram showed an ejection fraction of 0.20. Vital signs now are temperature 38.0°C (100.4°F), pulse 90/min, respirations 16/min, and blood pressure 130/90 mm Hg. Pulse oximetry on room air shows an oxygen saturation of 96%. Physical examination discloses no unexpected abnormalities. Which of the following regimens for prophylaxis of deep venous thrombosis will likely be most beneficial in this patient? (A) Continuous application of bilateral lower extremity pneumatic compression devices (B) Continuous intravenous infusion of heparin titrated to a PTT of 1.5 to 2.0 times the control value (C) Oral warfarin (D) Subcutaneous enoxaparin (E) Subcutaneous heparin]

**AI_E:** RESTATING THE QUESTION: We are considering the best method of deep venous thrombosis (DVT) prevention for a 72-year-old man who has recently had a left total knee replacement. DVT refers to the formation of a blood clot in the deep veins, typically of the legs, which can lead to serious complications like pulmonary embolism if left untreated. The patient's medical history includes coronary artery disease and his current conditions and medications suggest a compromised cardiovascular status. PLANNING: The patient is at a high risk for DVT due to his recent surgery and age. Some factors we need to consider include the patient's cardiovascular status, bleeding risk, and potential systemic effects of the prophylactic method. Each of the proposed prophylactic methods has different benefits and risks: mechanical methods (like pneumatic compression devices) pose less systemic risks, while anticoagulants (like heparin and enoxaparin) are more effective but pose higher systemic risks and require monitoring. CLINICAL REASONING: The patient's compromised cardiovascular status and the use of a fentanyl pump could increase the risk of complications with certain anticoagulants. Therefore, it's important to consider both the effectiveness of the prophylaxis against DVT and its safety for the patient. CONCLUDING: Given the patient's current clinical context, it appears that a balance between effectiveness and safety would be necessary. Therefore, both pharmacological and mechanical methods could be considered, depending on their risk and benefit evaluation. RESPONSE: In this context, the continuous application of bilateral lower extremity pneumatic compression devices (Option A) could be a beneficial regimen for prophylaxis of DVT. While this mechanical method may have lesser efficacy compared to pharmacological methods, it offers safety by avoiding potential systemic complications associated with anticoagulant therapy. However, close monitoring of the patient for any signs of DVT would be essential.

**AI_A:** The scenario presented involves identifying the most appropriate mode of prophylaxis against deep venous thrombosis (DVT) for a 72-year-old male patient who recently underwent a total knee replacement. The patient has notable previous medical history including coronary artery disease and has an ejection fraction of 0.20 which indicates heart's diminished ability to pump blood effectively. We're also given five possible regimens for prophylaxis against DVT; these include mechanical prevention measures like pneumatic compression devices, and pharmaceutical measures such as oral or injected anticoagulants. In terms of planning, the priority would be to prevent DVT while also avoiding any potential complications from the respective prophylaxis measures. The main assumptions we need to assess are the relative risks of DVT and prophylaxis-related complications in each scenario. We should consider the patient's compromised cardiovascular status and the risk of hemorrhage associated with anticoagulants. Given the patient's postoperative status and his compromised cardiovascular profile, the benefit-to-risk balance of each option should be evaluated carefully. Mechanical prophylaxis with bilateral lower extremity pneumatic compression devices would be effective in promoting venous return and prevent stagnation of blood, a risk factor for DVT. However, it's less effective than pharmacological prophylaxis. If any anticoagulant is selected for this patient, given his current state and medication regimen, we should consider the potential side effects and interactions that these drugs might have with the patient's current medicines. So, in conclusion, balancing the need for effective prophylaxis and the risk of potential adverse effects brought about by anticoagulation therapy, considering the patient's current medical condition, and keeping in mind the complications and logistics associated with monitoring of anticoagulation suggests that method (A), the continuous application of bilateral lower extremity pneumatic compression devices, would be the most beneficial for this patient. The response is (A): Continuous application of bilateral lower extremity pneumatic compression devices.

**AI_D:** In the presented clinical case, we are challenged to determine the most beneficial prophylactic strategy against deep venous thrombosis (DVT) for a 72-year-old male patient. The patient has just undergone a left total knee replacement and presents with various medical conditions such as coronary artery disease. He is receiving relief for his pain with a systemic patient-controlled analgesic pump delivering fentanyl. We are offered a selection of options to consider, which includes different medications and the application of pneumatic compression devices. It's important to clarify what deep venous thrombosis (DVT) is before we proceed. DVT refers to formation of a blood clot in one of the deep veins, typically in the legs. Given that the patient recently underwent knee replacement surgery, he is at increased risk of developing DVT. If not prevented, the clot could break loose, move through the bloodstream and lodge in the lungs, causing a potentially fatal pulmonary embolism. In evaluating the given options, I must point out that the patient's compromised cardiac condition could potentially limit the use of anticoagulants due to their systemic effects and bleeding risks. Given that his ejection fraction is just 0.20, his heart’s pumping ability is markedly below a healthy heart, which typically has an ejection fraction of 55% to 70%. This situation means that he would be more susceptible to cardiac complications brought on by systemic disruptions such as systemic anticoagulation. Given these variables, it’s plausible to consider continuous application of bilateral lower extremity pneumatic compression devices as a safer option, as it can provide mechanical DVT prevention without the systemic effects of anticoagulants. However, this would be less effective than pharmacological interventions. Another hypothesis worth considering is that systemic anticoagulants could still be used, but their dosage and monitoring need to be strictly controlled. Subcutaneous administration of low molecular weight heparins, like enoxaparin, could be a viable alternative. They have a more predictable anticoagulant response, lower risk of bleeding, and requires less monitoring compared to oral warfarin or continuously infused heparin. After considering the above premises and the patient’s overall clinical context, it appears that utilizing subcutaneous enoxaparin may be the most beneficial approach for DVT prophylaxis in this patient. It strikes a balance between DVT prevention efficacy and the patient's potential risks from anticoagulation therapy, factoring in his compromised cardiac condition. Therefore, from a clinical thought process, the conclusion points to subcutaneous enoxaparin (Option D) as the most likely beneficial regimen for DVT prophylaxis in this patient. But this should be confirmed with further clinical observations and tests and tailored according to changes in the patient's condition and response. To answer the posed question, the most beneficial regimen for DVT prophylaxis in the patient would likely be: (D) Subcutaneous enoxaparin.

**AI_C:** Restating the question: This scenario presents a 72-year old male patient who just underwent a total knee replacement procedure. The key objective here is to determine the most beneficial method for prophylaxis of deep venous thrombosis (DVT)— a blood clot that forms within the deep veins — usually in the legs. The options include mechanical (pneumatic compression devices) and pharmacological (continuous IV heparin, oral warfarin, subcutaneous enoxaparin, and subcutaneous heparin) methods. Plan: The assumption here is that the patient, post-knee replacement, is at risk for developing DVT. It is also assumed that his cardiovascular status is fragile considering his ejection fraction of 0.20 and the range of medications for the coronary artery disease. The alternate assumption is, therefore, that a regimen prioritizing safety over efficacy may be wiser, given the patient's condition. This would mean choosing a mechanical over pharmaceutical prophylaxis method. Clinical Reasoning: Let's examine the various prophylaxis methods proposed. Continuous application of bilateral lower extremity pneumatic compression devices (Option A) provides mechanical prophylaxis against DVT formation and is safer especially for patients with a precarious cardiovascular status. IV heparin (Option B) requires coagulation monitoring, which could complicate management given the patient's cardiac status. Warfarin, an oral anticoagulant (Option C), is inappropriate, given the immediate post-operative state of the patient. It also interacts with many drugs and requires monitoring to maintain an appropriate therapeutic range. Subcutaneous low molecular weight heparin like enoxaparin (Option D), and unfractionated heparin (Option E) offer effective prophylaxis but carry a bleeding risk, especially in a patient with cardiovascular diseases. Conclusion: Given the clinical context of our patient, the most beneficial approach would be a regimen that provides effective prophylaxis but prioritizes the safety of our patient, considering his diminished cardiac status. Response: I would recommend the continuous application of bilateral lower extremity pneumatic compression devices (Option A) as the most beneficial regimen for prophylaxis of deep venous thrombosis in this patient. The rationale for this choice centers around balancing the need for prophylaxis against DVT and prioritizing patient safety given his fragile cardiovascular state. Notwithstanding, for optimal results, any changes in the patient's status should warrant a revisit of this choice.

**AI_B:** The question asks us to identify the most suitable regimen for preventing deep venous thrombosis (DVT) in a 72-year-old patient who has just undergone a total knee replacement. This patient is being monitored in the intensive care unit for pain management and for ventricular ectopy due to a significant cardiac history, including coronary artery disease. The patient's ejection fraction is lower than the normal range (0.52-0.72 in males), indicating compromised heart function. Therefore, any medical decisions should take into consideration the patient's critical cardiac condition. Underlying our decision-making are some crucial medical facts to consider. Firstly, we know that orthopedic surgery patients are at a higher risk of developing a DVT, and pharmacological thromboprophylactic treatments, such as oral warfarin, subcutaneous heparin, and low-molecular-weight heparin (LMWH) like enoxaparin, can help mitigate this risk. Secondly, the use of these anticoagulants might be associated with an increased risk of bleeding, which is crucial considering our patient's pre-existing cardiac pathologies. The efficacy of these drugs also requires close monitoring, which could be challenging in an ICU setting. Using these facts, we could hypothesize that either mechanical prophylaxis methods or the use of a pharmacological agent with a lower risk of bleeding complications and less demanding monitoring requirements may be the suitable choices. As such, continuous application of bilateral lower extremity pneumatic compression devices (option A) and subcutaneous enoxaparin (option D) can be considered as the main alternatives. Considering the patient's poor ejection fraction and the associated increased risk of bleeding, mechanical prophylaxis could be seen as favorable due to its non-systemic nature. However, LMWH like enoxaparin might be the better choice due to its proven efficacy in reducing the risk of DVT, lower bleeding risk compared to unfractionated heparin, and less demanding monitoring schedule. Reviewing these assumptions and applying them to our patient, it's crucial to balance both the effectiveness of DVT prophylaxis and the safety associated with the given therapies. While pneumatic compression devices give a non-systemic approach with reduced risk of bleeding, their effectiveness might not be equivalent to pharmacological therapies. Enoxaparin, on the other hand, can provide stronger prophylaxis with manageable bleeding risk and avoids a strict monitoring schedule, better fitting our patient's critical condition and ICU setting. So, in this case, the correct answer would be (D) Subcutaneous enoxaparin. This choice is derived from reasoning that takes into consideration the patient's high risk of DVT, the critical cardiac condition, and the challenges associated with the ICU setting. The use of enoxaparin would balance the need to efficiently prevent DVT while managing the associated bleeding risk and monitoring requirements.

**Facilitator** **[Round 4]:** Summary: AI_A, AI_C, and AI_E recommend the continuous application of bilateral lower extremity pneumatic compression devices (Option A) as the most beneficial regimen for DVT prophylaxis in the patient. They argue that this mechanical method, while less effective than pharmacological methods, is safer given the patient's compromised cardiovascular status and the potential systemic complications associated with anticoagulant therapy. On the other hand, AI_B and AI_D suggest subcutaneous enoxaparin (Option D) as the most beneficial regimen. They argue that while this pharmacological method carries a risk of bleeding, it is more effective in preventing DVT. They also note that enoxaparin has a lower bleeding risk compared to other anticoagulants and requires less monitoring, making it a suitable choice given the patient's critical condition and ICU setting. Key Differences: The key differences in the council members' perspectives lie in their evaluation of the balance between the effectiveness of DVT prophylaxis and the safety of the patient. AI_A, AI_C, and AI_E prioritize safety over efficacy, recommending a mechanical method that avoids potential systemic complications. In contrast, AI_B and AI_D prioritize efficacy, recommending a pharmacological method that is more effective in preventing DVT, while still considering the patient's safety by choosing a drug with a manageable bleeding risk and less demanding monitoring requirements. Question to Clarify Disagreements: Given the patient's compromised cardiovascular status and the potential systemic complications associated with anticoagulant therapy, how do you weigh the importance of safety versus efficacy in choosing the most beneficial regimen for DVT prophylaxis? Can you provide more insight into your decision-making process, particularly regarding the balance between the effectiveness of DVT prophylaxis and the safety of the patient?. Based on your preceding response, how would you answer the original question: [A 72-year-old man is admitted to the intensive care unit for pain management and observation of ventricular ectopy 4 hours after undergoing uncomplicated left total knee replacement. He is receiving fentanyl via systemic patient-controlled analgesic pump for pain management. Medical history is remarkable for coronary artery disease, for which he takes atorvastatin, furosemide, carvedilol, lisinopril, and 81-mg aspirin. Baseline echocardiogram showed an ejection fraction of 0.20. Vital signs now are temperature 38.0°C (100.4°F), pulse 90/min, respirations 16/min, and blood pressure 130/90 mm Hg. Pulse oximetry on room air shows an oxygen saturation of 96%. Physical examination discloses no unexpected abnormalities. Which of the following regimens for prophylaxis of deep venous thrombosis will likely be most beneficial in this patient? (A) Continuous application of bilateral lower extremity pneumatic compression devices (B) Continuous intravenous infusion of heparin titrated to a PTT of 1.5 to 2.0 times the control value (C) Oral warfarin (D) Subcutaneous enoxaparin (E) Subcutaneous heparin]

**AI_E:** Taking a closer look at the question, a 72-year-old male patient with coronary artery disease, who recently underwent a total knee replacement and is under pain management for ventricular ectopy, is inquiring about the most beneficial regimen for preventing deep venous thrombosis (DVT). This condition, DVT, is a formation of a thrombus (clot) in the deep veins, commonly in the lower limbs—which is a serious potential complication after surgery. The prophylaxis options given range from mechanical (i.e., pneumatic compression devices) to pharmacological measures (e.g., heparin, warfarin, enoxaparin). To answer this question, the assumptions we're working with are: (1) the patient's existing medical condition (coronary artery disease and low ejection fraction) could heighten the risk of anticoagulant-associated bleeding, and (2) postoperative period, patient's immobility and underlying cardiac condition might increase his risk of developing DVT. An alternate assumption could be that despite the risks that come with pharmacological prophylaxis, the possibility of DVT could also pose a life-threatening risk to the patient (e.g., pulmonary embolism). Therefore, the effectiveness of preventing DVT might override the potential risks of anticoagulant-associated bleeding, especially if the choice of pharmacological agent has a lower bleeding risk and less demanding monitoring requirements. Regarding clinical reasoning, it's critical to note that each prophylaxis strategy carries different risks and benefits. Mechanical methods, like pneumatic compression devices, are prone to be less effective compared to pharmacological methods but have the benefit of avoiding potential systemic complications. On the contrary, pharmacological methods, such as enoxaparin, are generally more effective in preventing DVT, albeit at the expense of a potential increased risk for bleeding. In conclusion, it is crucial to weigh the risk of bleeding due to pharmacological prophylaxis against the risk of DVT/PE. The best option would be the one that carries the least systemic risk to the patient while effectively preventing DVT. Considering the clinical scenario outlined: the patient's age, his cardiac condition, postoperative status, and the relative safety and efficacy of the different methods of DVT prophylaxis, We believe that the most beneficial regimen for prophylaxis of deep venous thrombosis in this patient would be the continuous application of bilateral lower extremity pneumatic compression devices (Option A). This method offers a lower-risk solution while addressing the need for DVT prophylaxis following the total knee replacement surgery.

**AI_B:** Restating the issue at hand, a decision is needed regarding prophylaxis of DVT in a 72-year-old male patient with significant medical history. The patient has undergone left total knee replacement, and has substantial medical history factors including coronary artery disease, furosemide, carvedilol, lisinopril, 81-mg aspirin, and low ejection fraction. DVT prophylaxis options include mechanical or pharmacologic regimens, with nuances for each available modality. Mechanical prophylaxis, such as the use of bilateral lower extremity pneumatic compression devices minimizes blood clotting event risk without introducing the potential for systemic complications associated with anticoagulants. On the other hand, pharmacological prophylaxis, such as the use of subcutaneous enoxaparin, can prevent DVT more effectively but carry a risk of bleeding and require monitoring. The alternate assumption generated here is that while safety is a primary concern, the clinical priority in this case should be the prevention of deep venous thrombosis (DVT) given the patient's postoperative state and notable risk factors including advanced age and cardiac history. In exploring the question of safety versus efficacy, let's consider the clinical implications. Patients recovering from total knee replacement surgery are at a heightened risk for DVT due to immobility and vascular endothelial injury. Therefore the efficacy of DVT prevention plays a vital role. This must be balanced with safety considerations, especially in patients with significant cardiac histories and on multiple medications, where additional pharmacotherapy could introduce more systemic complications. Considering this, subcutaneous enoxaparin (Option D) might be viewed as a strong candidate. This low molecular weight heparin (LMWH) is an effective prophylaxis for DVT and generally requires less laboratory monitoring than other anticoagulation agents such as warfarin or unfractionated heparin. While it does bring a risk of bleeding, LMWHs have been found to pose a lower bleeding risk than other anticoagulants. In conclusion, while recognizing the importance of safety concerns, particularly within this patient's specific clinical context, the emphasis on high efficacy DVT prophylaxis leads to the recommendation of subcutaneous enoxaparin. This regimen not only provides substantial protection against DVT but also poses a relatively lower risk of bleeding compared to other anticoagulants. Accordingly, my response is: (D) Subcutaneous enoxaparin.

**AI_A:** This scenario concerns a 72-year-old man who has been admitted to the intensive care unit (ICU) for observation due to signs of ventricular ectopy and pain management after a knee replacement surgery. His medical history involves coronary artery disease and he has been on medication for the same. His vitals seem stable. We are asked to identify the most beneficial regimen for deep venous thrombosis (DVT) prevention. DVT is a serious condition where blood clots form in the deep veins, typically in the lower legs, thighs, or pelvis. Several factors can increase the risk of DVT, including surgery (especially orthopedic), immobility, advanced age and certain health conditions such as malignancies and cardiovascular disease. In thinking about the patient, we need to consider several assumptions including his age, his surgery, his cardiac function, his pain medication, and his ventricular ectopy. Of these assumptions, the ones with potential clinical implication include his surgery, underlying heart disease and post-surgical mobility, which put him at risk of developing DVT. Therefore, considering all the contributing factors, the primary goal in the immediate post-operative period should be to prevent the occurrence of DVT using physical means, medication, or a combination of the two, depending on the patient's overall condition and risk levels. Mechanical prophylaxis, in the form of lower extremity pneumatic compression devices, can be used alone or in combination with medication for patients at risk of DVT in the post-operative period. Anticoagulant medications, such as heparin, enoxaparin, and warfarin, prevent the formation of new clots and reduce the risk of existing clots getting larger. For this particular patient, his impaired cardiac function due to coronary artery disease, as indicated by an ejection fraction of 0.20, should be considered while formulating a preventive strategy for DVT. Anticoagulants, despite being more effective at preventing DVT, carry a risk of causing bleeding, which can have serious, even fatal, consequences especially in someone with compromised cardiovascular function. Given these factors, my final response would be that given the risk associated with our patient, the most beneficial regimen to prevent DVT would be the continuous application of bilateral lower extremity pneumatic compression devices (Option A). These devices can provide prophylaxis against DVT without the bleeding risk associated with anticoagulant therapy. Their use is particularly beneficial to patients like the one in question, who has compromised cardiovascular function and is in pain, limiting his mobility. Initially, the safety of the patient takes precedence. However, the situation would need to be continuously monitored and reassessed for the need for additional measures.

**AI_D:** RESTATING THE QUESTION: In simpler terms, we need to decide on the most effective form of deep venous thrombosis (DVT) prevention for a 72-year-old man who just had knee replacement surgery. DVT is a blood clot that forms in a vein deep in the body, typically the legs, which can lead to complications if it travels to the lungs (pulmonary embolism). This patient has notable cardiac risk factors, such as low ejection fraction and coronary artery disease. The question presents several DVT prevention techniques - mechanical (compression devices) and pharmacological (various blood thinners). PLANNING: There are several assumptions here. Firstly, we assume that the patient has a high risk of developing DVT due to his postoperative state and immobility, which is a major risk factor for DVT. We also need to consider his cardiac condition, which may make the use of blood thinners hazardous due to the potential for bleeding complications. Thus, we must weigh the need for effective DVT prevention versus the potential cardiovascular risks associated with the pharmacological options. CLINICAL REASONING: We must consider the risk-benefit ratio for this patient. On the one hand, we have the risk of DVT due to surgery and immobility. On the other, there is a risk of bleeding complications and additional strain on the heart with the pharmacological options. Compression devices would be effective at promoting circulation and reducing the risk of DVT without any additional strain on the heart or risk of bleeding. However, the pharmacological options are generally more effective at preventing DVT, although they present a greater risk of complications, including bleeding and potential strain on the heart, particularly in a patient with a lower ejection fraction. Enoxaparin, a low molecular weight heparin, offers effective prevention for DVT with a lower risk for bleeding compared to unfractionated heparin and needs less monitoring, making it a suitable choice for an ICU patient. CONCLUDING: When looking at the balance of efficacy and safety in this situation, it seems that while mechanical prevention has the least risk, the pharmacological prevention with Subcutaneous enoxaparin (D) provides a higher level of efficacy with an acceptable safety profile in the context of this patient's cardiac condition. RESPONSE: The most beneficial regimen for DVT prophylaxis in this patient would likely be Subcutaneous enoxaparin (Option D). Despite some cardiac risk factors, the increased risk of clot formation due to his postoperative state warrants the use of a more effective form of DVT prevention. Enoxaparin provides this level of protection while still carrying a manageable risk profile relative to the other anticoagulant options.

**AI_C:** The question is asking us to determine the most beneficial method for the prevention of deep vein thrombosis (DVT) in a 72-year-old male patient who underwent left total knee replacement surgery and is currently in the intensive care unit (ICU) for pain management and observation of ventricular ectopic activity. The patient's medical history includes coronary artery disease (CAD) and he is presently experiencing a fever. His current medications include atorvastatin, furosemide, carvedilol, lisinopril, and an 81-mg aspirin daily. His echocardiogram shows a reduced ejection fraction of 0.20, signifying a weak heart pump function. His vitals are normal except for a slightly elevated temperature. Deep vein thrombosis (DVT) is a serious condition in which a blood clot forms in one of the deep veins of the body, typically the legs. This condition is especially concerning in postoperative patients, as the combination of the patient's immobility and alterations in the balance between clot formation and dissolution can lead to thrombosis. There are various prophylactic strategies for DVT, including mechanical methods (e.g., intermittent pneumatic compression devices), and pharmacologic methods (e.g., heparin, warfarin, enoxaparin). Here, the assumptions are that the regimensB, C, D, and E (Heparin IV, Warfarin, Enoxaparin, and Heparin SC respectively) will be more effective in preventing DVT, but they also carry a risk with systemic effects, particularly bleeding complications. The advantage of regimen A (pneumatic compression devices) is that it does not carry the risk of systemic effects, but is less effective. Using a clinical reasoning process, considering the patient's medical history of coronary artery disease and his weak heart pump function indicated by an ejection fraction of 0.20, we need to be careful in choosing a prophylactic regimen that would not add further strain on his cardiovascular system. Pharmacological DVT prophylactic regimens, such as IV heparin, oral warfarin, subcutaneous enoxaparin, or heparin, carry risks predominantly related to bleeding complications, especially in patients with compromised cardiovascular systems. On the other hand, mechanical methods like pneumatic compression devices are safer but less effective in DVT prophylaxis. Concluding, the non-pharmacological method of applying bilateral lower extremity pneumatic compression devices is likely the most beneficial method for DVT prophylaxis in this patient. This method ensures that he is not exposed to the systemic risks associated with anticoagulant therapy, particularly bleeding complications given his compromised cardiovascular state. Although it is less effective than pharmacological methods, safety should be clinically prioritized in this case. Therefore, to the posed question; the most beneficial method for prophylactic prevention of deep venous thrombosis in this patient would be the continuous application of bilateral lower extremity pneumatic compression devices (Option A).

**Facilitator** **[Round 5]:** Summary: AI_A, AI_C, and AI_E believe that the most beneficial regimen for DVT prevention in the patient would be the continuous application of bilateral lower extremity pneumatic compression devices (Option A). They argue that this method provides prophylaxis against DVT without the bleeding risk associated with anticoagulant therapy, which is particularly beneficial for patients with compromised cardiovascular function. They emphasize the importance of safety and the need to avoid additional strain on the patient's cardiovascular system. On the other hand, AI_B and AI_D recommend subcutaneous enoxaparin (Option D) as the most beneficial regimen for DVT prophylaxis. They argue that while safety is important, the clinical priority should be the prevention of DVT given the patient's postoperative state and notable risk factors. They believe that enoxaparin provides substantial protection against DVT and poses a relatively lower risk of bleeding compared to other anticoagulants. Key Differences: The key differences in the council members' perspectives lie in their prioritization of safety versus efficacy. AI_A, AI_C, and AI_E prioritize safety and minimizing risk, particularly given the patient's compromised cardiovascular function. They believe that mechanical prophylaxis, despite being less effective, is the safer option. In contrast, AI_B and AI_D prioritize efficacy in preventing DVT, arguing that the patient's postoperative state and risk factors necessitate a more effective prophylactic regimen, despite the associated risks. Question for Council Members: Given the differing perspectives, a clarifying question could be: "How do you weigh the importance of safety versus efficacy in this specific case, and could a combination of mechanical and pharmacological prophylaxis be considered to balance both safety and efficacy?". Based on your preceding response, how would you answer the original question: [A 72-year-old man is admitted to the intensive care unit for pain management and observation of ventricular ectopy 4 hours after undergoing uncomplicated left total knee replacement. He is receiving fentanyl via systemic patient-controlled analgesic pump for pain management. Medical history is remarkable for coronary artery disease, for which he takes atorvastatin, furosemide, carvedilol, lisinopril, and 81-mg aspirin. Baseline echocardiogram showed an ejection fraction of 0.20. Vital signs now are temperature 38.0°C (100.4°F), pulse 90/min, respirations 16/min, and blood pressure 130/90 mm Hg. Pulse oximetry on room air shows an oxygen saturation of 96%. Physical examination discloses no unexpected abnormalities. Which of the following regimens for prophylaxis of deep venous thrombosis will likely be most beneficial in this patient? (A) Continuous application of bilateral lower extremity pneumatic compression devices (B) Continuous intravenous infusion of heparin titrated to a PTT of 1.5 to 2.0 times the control value (C) Oral warfarin (D) Subcutaneous enoxaparin (E) Subcutaneous heparin]

**AI_A:** 1) RESTATING THE QUESTION: A 72-year-old man who had recently undergone an uncomplicated left knee replacement is now in the ICU for pain management and observation of ventricular ectopy. He has a known history of coronary artery disease (CAD), which contributes to his low ejection fraction (0.20), indicative of reduced heart function. His current treatment involves various medications for CAD and fentanyl for pain management from a patient-controlled analgesic pump. Given his condition and recent surgical procedure, it is crucial to decide on a regimen for prophylaxis of deep venous thrombosis (DVT), which refers to the formation of a blood clot in the deep veins, commonly in the lower limbs. 2) PLANNING: This decision involves safety and effectiveness considerations. The patient's low ejection fraction and CAD pose potential complications with most forms of DVT prophylaxis, which typically involve anticoagulants. Anticoagulants present potential bleeding risks, which could further complicate his cardiac condition. Therefore, I hypothesize that avoiding treatments with such risks should be prioritized in this situation. 3) CLINICAL REASONING: The options presented are: (A) Pneumatic compression devices on both lower extremities, (B) Continuous intravenous heparin infusion, (C) Oral warfarin, (D) Subcutaneous enoxaparin, and (E) Subcutaneous heparin. The anticoagulants (B, C, D, E) carry bleeding risk, which may strain the patient's cardiovascular system further. In contrast, pneumatic compression devices (A) mechanically prevent blood pooling and clot formation in the deep veins without bleeding risk. 4) CONCLUDING: Given the patient's cardiac history and current condition, I propose that the safest and likely the most beneficial approach would be to opt for a non-pharmaceutical intervention, specifically pneumatic compression devices. 5) RESPONSE: Therefore, I believe that the patient would most benefit from the continuous application of bilateral lower extremity pneumatic compression devices (Option A). This choice balances the efficacy in preventing DVT and ensuring patient safety, primarily avoiding unnecessary strain on his cardiovascular system.

**AI_C:** Restating the Question: The question revolves around a 72-year-old man who recently underwent left total knee replacement and is admitted to the ICU for pain management and observation of ventricular ectopy. Given his medical history of coronary artery disease and current cardiovascular status, we're seeking the most beneficial regimen for prophylaxis against deep venous thrombosis (DVT), a condition that involves a blood clot in a deep vein, most commonly in the leg. Planning: The fundamental assumption here is that all postoperative patients, particularly those undergoing orthopedic procedures, are at an elevated risk for DVT due to factors like immobility, hypercoagulable state, and vascular injury. However, different prophylactic methods carry their own risks and benefits, and these must be weighed appropriately in the context of the patient's history and current health status. The question, then, is which DVT prophylaxis method would be most beneficial since the patient already has an evident cardiovascular compromise? Clinical Reasoning: DVT prophylaxis in postoperative patients can be approached with either pharmacological or mechanical methods, or a combination of both. Pharmacological methods include anticoagulants, which thin the blood to prevent clotting, but they can increase the risk of bleeding, particularly problematic in patients with compromised cardiovascular systems such as this patient. Mechanical methods, on the other hand, like pneumatic compression devices, promote blood circulation to prevent blood from pooling and clotting, but they may not be as effective as pharmacological methods. In this patient's case, his compromised ejection fraction, a marker for a reduction in the heart's ability to pump out blood, is a concern. Also, his coronary artery disease puts him at higher risk for major bleeding with anticoagulants. Therefore, it seems reasonable to prioritize safety over efficacy and select a method that minimizes risk, such as pneumatic compression devices. Concluding: Considering everything, while the pharmacological methods may be more effective, the mechanical method is safer for this patient due to his cardiovascular compromise. Therefore, without undermining the arguments for the use of pharmacologic agents in DVT prophylaxis, particularly in postoperative patients, for this patient under his current conditions, the use of mechanical prophylaxis in the form of a continuous application of bilateral lower extremity pneumatic compression devices may be the most beneficial. Response: So, my response to the question is: (A) Continuous application of bilateral lower extremity pneumatic compression devices.

**AI_E:** Restating the Question: The question is asking about the ideal method of deep venous thrombosis (DVT) prophylaxis in a 72-year-old man who recently underwent left total knee replacement surgery. This patient has a significant medical history, including advanced coronary artery disease (CAD), which could increase his risk of complications from certain DVT prophylaxis methods. Deep venous thrombosis is a condition in which blood clots form in the deep veins, most commonly of the leg. Without prophylaxis, the risk of DVT increases especially after surgeries such as total knee replacement. DVT can lead to serious complications like pulmonary embolism, which can be life-threatening. Planning: The assumptions here involve the efficacy and safety of various methods of DVT prophylaxis. The alternate assumption challenges the safety of pharmacological prophylaxis in this patient due to his history of CAD and compromised cardiac function. What if we prioritized the safety over the efficacy of prophylaxis due to his compromised cardiovascular system? Clinical Reasoning: Given the patient's low ejection fraction, history of CAD and his recent surgery, he is at a high risk of both DVT and bleeding. Therefore, while he needs potent DVT prophylaxis, his risk of bleeding may be increased with certain methods. Prophylactic methods like subcutaneous enoxaparin (Option D) or heparin (Option E) may provide potent DVT protection but might increase the risk of bleeding, potentially worsening the patient's CAD and compromising his cardiac health. Alternatively, mechanical methods such as continuous application of bilateral lower extremity pneumatic compression devices (Option A) might pose a lower risk of bleeding but could also be less effective in preventing DVT. Concluding: After careful consideration of the patient's risks and benefits, the best choice might be a method that balances both safety and efficacy. In this case, the continuous application of bilateral lower extremity pneumatic compression devices might serve as a preferred prophylactic method due to its minimal risk of bleeding and potential to offer some degree of DVT protection. Response: The most beneficial regimen for prophylaxis of deep venous thrombosis in this patient would likely be (A) Continuous application of bilateral lower extremity pneumatic compression devices. This method may provide some DVT prophylaxis without posing additional bleeding risk, which should be kept to a minimum given the patient's cardiovascular status.

**AI_B:** RESTATING THE QUESTION: The patient, a 72-year-old man with a significant cardiovascular medical history, has just undergone a left total knee replacement. We are tasked with determining the most beneficial prophylaxis regimen for deep venous thrombosis (DVT) in his case, given the options of bilateral lower extremity pneumatic compression devices, intravenous heparin infusion, oral warfarin, subcutaneous enoxaparin, or subcutaneous heparin. PLANNING: Mechanistic reasoning hints towards the subcutaneous enoxaparin, as it's a type of low-molecular-weight heparin, often preferred due to its better bioavailability and longer half-life compared to unfractionated heparin. Furthermore, enoxaparin doesn't require monitoring of coagulation parameters. However, this patient's cardiovascular history, particularly the compromised cardiac function (ejection fraction of 0.20), requires careful consideration of the bleeding risk. CLINICAL REASONING: Major surgery, including total knee replacement, predisposes patients to DVT due to post-operative immobility and venous stasis. Pharmacological prophylaxis with anticoagulants, often in combination with mechanical methods, can reduce that risk. However, the decision about the type of anticoagulant often depends on the patient's overall health status, potential for drug-drug interactions, and bleeding risk. This individual's extensive cardiac disease with reduced ejection fraction and concomitant cardiovascular medications necessitate a cautious approach to avoid further straining the cardiovascular system. On the other hand, the significant risk of DVT warrants a reliable method of prophylaxis. CONCLUDING: It seems a balanced approach would be the most beneficial. The bilateral lower extremity pneumatic compression devices would help reduce stasis and thus prevent thrombosis while avoiding the risk of bleeding. However, they might not provide adequate prophylaxis alone for a high-risk situation like post knee-replacement surgery in old age. Hence, a pharmacological agent with a balanced safety and efficacy profile would complement pneumatics well. In this context, subcutaneous enoxaparin might suit the need as it is highly efficacious and has a relatively lower bleeding risk. RESPONSE: Considering the clinical priority of preventing DVT and keeping the safety aspect of cardiovascular robustness in mind, I would vote for subcutaneous enoxaparin (Option D). A combination with intermittent pneumatic compression devices as an adjunct would further mitigate risk.

**AI_D:** 1) RESTATING THE QUESTION: We are discussing a 72-year-old man who has recently undergone a left total knee replacement and is being monitored in the intensive care unit. His notable medical history includes coronary artery disease and an exceptionally low ejection fraction. The question asks which regimen would most beneficially prevent deep venous thrombosis (DVT), a serious potential complication after surgery, in this patient. 2) PLANNING: Key assumptions include the patient's surgical status, cardiovascular condition, and his overall risk profile, which includes the presence of coronary artery disease and a significantly reduced ejection fraction. The appropriateness of pharmacological prophylaxis (such as heparin or enoxaparin) is directly informed by these assumptions. As an alternative assumption, we could question whether mechanical methods of DVT prophylaxis (such as pneumatic compression devices) might be more appropriate given the patient's compromised cardiac function and his current pain management regimen, which includes the opioid furosemide. With opioid usage, there is the possibility of a pruritus-induced scratching, potentially leading to bleeding with anticoagulation therapy. 3) CLINICAL REASONING: Given the patient's compromised cardiovascular function, the risk of bleeding with anticoagulation therapy is a significant concern. Pneumatic compression devices may provide a pragmatic alternative: they lower the risk of DVT without subjecting the patient to bleeding risks that might further compromise his cardiovascular health. Yet, the thrombosis risk after joint replacement surgery is substantial and the pneumatic compression devices provide relatively less certain protection against DVT. The patient's cardiovascular health is already compromised and a DVT could potentially lead to a pulmonary embolism, a life-threatening complication. Enoxaparin, in contrast to pneumatic compression devices, provides more reliable DVT prevention. Though it carries with it a risk of bleeding, with careful management, this risk can be mitigated and might be a reasonable trade-off considering the substantial risk of DVT in the patient's current state. 4) CONCLUDING: Evaluation of the potential options for DVT prophylaxis in this patient suggests that while both mechanical and pharmacological methods have their own merits and risks, consideration of the patient's overall health status may favor pharmacological intervention with enoxaparin. 5) RESPONSE: I would therefore recommend subcutaneous enoxaparin (Option D) as the most beneficial regimen for preventing deep venous thrombosis in this patient. This takes into account both the immediate surgical status of the patient, the high risk for DVT in the postoperative period, and the associated risks of each method given the patient's pre-existing medical conditions. Notably, this approach seeks to balance both efficacy and safety, protecting the patient from DVT while remaining aware of the inherent risks associated with each prophylactic strategy.

**Facilitator** **[Round 6]:** Summary: AI_A, AI_C, and AI_E all recommend the use of pneumatic compression devices (Option A) for DVT prophylaxis in the patient. They argue that given the patient's compromised cardiovascular health, the risk of bleeding associated with anticoagulants outweighs their potential benefits. They believe that the mechanical method of pneumatic compression devices, while potentially less effective, is safer for this patient. On the other hand, AI_B and AI_D suggest the use of subcutaneous enoxaparin (Option D). They acknowledge the bleeding risk associated with anticoagulants but argue that the high risk of DVT in the postoperative period necessitates a more reliable method of prophylaxis. They believe that with careful management, the bleeding risk associated with enoxaparin can be mitigated. Question for Council Members: Given the patient's compromised cardiovascular health and the high risk of DVT in the postoperative period, how do you weigh the relative importance of safety and efficacy in choosing a method of DVT prophylaxis? How do you justify your choice of prophylaxis in light of the potential risks and benefits associated with each method?. Based on your preceding response, how would you answer the original question: [A 72-year-old man is admitted to the intensive care unit for pain management and observation of ventricular ectopy 4 hours after undergoing uncomplicated left total knee replacement. He is receiving fentanyl via systemic patient-controlled analgesic pump for pain management. Medical history is remarkable for coronary artery disease, for which he takes atorvastatin, furosemide, carvedilol, lisinopril, and 81-mg aspirin. Baseline echocardiogram showed an ejection fraction of 0.20. Vital signs now are temperature 38.0°C (100.4°F), pulse 90/min, respirations 16/min, and blood pressure 130/90 mm Hg. Pulse oximetry on room air shows an oxygen saturation of 96%. Physical examination discloses no unexpected abnormalities. Which of the following regimens for prophylaxis of deep venous thrombosis will likely be most beneficial in this patient? (A) Continuous application of bilateral lower extremity pneumatic compression devices (B) Continuous intravenous infusion of heparin titrated to a PTT of 1.5 to 2.0 times the control value (C) Oral warfarin (D) Subcutaneous enoxaparin (E) Subcutaneous heparin]

**AI_E:** RESTATING THE QUESTION: This is a clinical case involving a 72-year-old man with cardiovascular comorbidities who has recently had a total knee replacement surgery. The main query relates to determining the most effective and safe regimen for the prophylaxis of deep venous thrombosis (DVT). DVT is a condition in which a blood clot forms in one of the deep veins, usually in the leg. As patients undergoing major orthopedic surgeries, such as total knee replacement, have a high risk of developing DVT, prophylactic measures are recommended. PLANNING: The critical assumptions are that anticoagulants have a significant risk of bleeding, particularly in this patient with cardiovascular comorbidities and that mechanical devices can lower the risk of DVT without this bleeding risk. To evaluate these assumptions, I will consider the effectiveness and safety of both pharmacological and mechanical strategies used for DVT prophylaxis after surgery. CLINICAL REASONING: Prophylaxis for DVT in postoperative patients, especially those undergoing total knee replacement surgeries, is critical due to the inherent high risk. Various prophylactic strategies can be classified into two primary groups: anticoagulant therapies, such as heparin and warfarin, and mechanical methods, such as compression devices. While anticoagulant therapies are generally effective, they come with a significant risk of bleeding, which in this patient with cardiovascular comorbidities could lead to adverse outcomes. On the other hand, mechanical methods like continuous pneumatic compression devices may be less efficient in preventing DVT, but they pose no risk of bleeding. CONCLUDING: When balancing safety and efficacy, mechanical methods appear to be the safer option for this patient considering his risk factors. The continuous application of bilateral lower extremity pneumatic compression devices would theoretically significantly lower his risk of bleeding-associated complications, while also aiding in DVT prophylaxis. RESPONSE: Hence, according to my clinical understanding, the continuous application of bilateral lower extremity pneumatic compression devices (Option A) seems to be the regimen that would likely be the most beneficial for this patient undergoing postoperative DVT prophylaxis.

**AI_C:** RESTATING THE QUESTION: The question asked is to determine the best prophylactic regimen against deep venous thrombosis (DVT) for a 72-year-old post-operative patient with compromised cardiovascular health. The patient has undergone a knee replacement operation and is currently under pain management in the intensive care unit. DVT is a blood clot that forms in a vein deep inside a part of the body and mainly affects the large veins in the lower leg and thigh. PLANNING: The critical assumptions here include the efficacy and potential side effects of each prophylactic measure, with the primary emphasis on the patient's significant cardiovascular history. I would question whether or not the patient's already compromised cardiovascular system could handle the potential bleeding risk posed by the anticoagulants listed in the options. CLINICAL REASONING: We must balance the effectiveness of each method against its safety, particularly in light of the patient's compromised cardiovascular health. The potential benefits of anticoagulants in preventing DVT must be weighed against their potential to cause bleeding, particularly for a patient with a history of coronary artery disease. Similarly, while mechanical methods such as pneumatic compression devices may be less effective, their safety profile may make them the better option for this patient. CONCLUDING: Given the patient's age, history of coronary artery disease, low ejection fraction (a measure of how much blood the left ventricle pumps out with each contraction), and the post-surgical state all increase the risk of both bleeding and thrombosis. A prophylaxis with a high efficacy but also high bleeding risk might not be appropriate for this patient. Therefore, I suggest we consider pneumatic compression devices (Option A) for this patient, despite its lesser efficacy. RESPONSE: As per the clinical reasoning and medical parameters of the patient, the most beneficial regimen for prophylaxis of DVT in this patient would be continuous application of bilateral lower extremity pneumatic compression devices (Option A). This mechanical method of prophylaxis reduces the chances of bleeding common with pharmacological prophylaxis, making it a safer option for this specific patient with a remarkable cardiovascular history.

**AI_A:** RESTATING THE QUESTION: The question asks about the best options for deep venous thrombosis (DVT) prophylaxis for a 72-year-old male patient who is 4 hours post-op after a left total knee replacement. He is in ICU, receiving pain management via a fentanyl pump and exhibiting other symptoms such as ventricular ectopy. This patient has a medical history of coronary artery disease and low ejection fraction (.20). The possible prophylaxis methods include pneumatic compression devices, IV heparin, oral warfarin, subcutaneous enoxaparin, and subcutaneous heparin. PLANNING: Key details to focus on include the patient's medical history of coronary artery disease and low ejection fraction, the fact that he has just undergone surgery, and the array of pharmaceutical options being considered for DVT prophylaxis. The big assumptions are that each method has its benefits and risks and that the most beneficial method would depend on the patient's specific condition and risk factors. Alternate assumptions might include questions about the effectiveness and safety for each method, particularly in light of this patient's individual context. CLINICAL REASONING: Total knee replacement surgery is a major risk factor for DVT due to venous stasis stemming from surgical manipulation and postoperative immobility. Additionally, this patient has a history of coronary artery disease and low ejection fraction, potentially increasing his risk of postoperative complications including DVT. While anticoagulants like heparin and enoxaparin can be beneficial in preventing DVT, they also carry a risk of bleeding, which can be especially concerning in a patient with a history of cardiac issues. Pneumatic compression devices provide a non-invasive method of DVT prophylaxis that doesn't carry the same risk of bleeding, but their effectiveness may be less when compared to pharmacological interventions. CONCLUDING: Considering both the patient's high risk of DVT and his existing cardiovascular issues, it appears essential to choose a prophylaxis regimen that would maximize efficacy while also minimizing risk. Ensuring safety is critical here due to the patient's coronary artery disease and low ejection fraction. In this scenario, it seems that managing bleeding risk might be a priority over maximizing efficacy in preventing DVT, and therefore a non-pharmacological approach may be more appropriate. RESPONSE: Based on the patient's condition and the stated criteria, I’d opt for option (A) Continuous application of bilateral lower extremity pneumatic compression devices, because it provides effective DVT prophylaxis while avoiding the potential bleeding risks associated with other pharmacological options.

**AI_B:** Restating the Question: The question is asking to decide what would be the most suitable treatment for preventing Deep Venous Thrombosis (DVT) in a 72-year-old male patient with significant cardiovascular health issues post knee surgery. The given options include various mechanical and pharmacological interventions: bilateral lower extremity pneumatic compression devices, a continuous intravenous infusion of heparin, oral warfarin, subcutaneous enoxaparin, and subcutaneous heparin. Planning: One primary consideration when choosing a prophylaxis measure for DVT is balancing the efficacy and risks associated with each method. Efficacy is evaluated based on how well the method can prevent DVT formation, while risks include bleeding, especially in patients with pre-existing cardiovascular issues, and other side effects. Another major concern should be the patient's current medications and their possible interactions with the chosen prophylaxis method. The options include non-pharmacological methods (pneumatic devices), which present minimal bleeding risk, and pharmacological methods (heparin, warfarin, enoxaparin), which provide robust prevention against DVT but present a significant bleeding risk. Clinical Reasoning: The patient has a history of coronary artery disease, indicating a compromised cardiovascular health status, and he is postoperative from a total knee replacement, placing him at a high risk of DVT. This necessitates a strong prophylactic strategy. Mechanical methods like the lower extremity pneumatic compression devices generally have less efficacy in DVT prophylaxis than pharmacological methods. However, they present negligible risk of bleeding, a crucial factor given the patient's cardiovascular status. On the other hand, pharmacological methods such as subcutaneous heparin or enoxaparin offer robust DVT prevention but increase the risk of bleeding. Given the high risk of DVT and the precarious cardiovascular condition of this patient, a more rigorous approach to DVT prevention might be warranted, even at the expense of increased risk of hemorrhage. Importantly, the potential hemorrhagic consequences can be monitored and managed in the controlled environment of the intensive care unit where the patient is currently admitted. Concluding: After considering the efficacy, risks, and patient's comorbidities, subcutaneous enoxaparin (Option D) may provide the best DVT prophylaxis for the patient. With the patient's compromised cardiovascular health and the increased risk of DVT post orthopedic surgery, a more effective measure than pneumatic compression devices is desirable. The risks associated with enoxaparin can be mitigated with proper monitoring and dose adjustment. Response: On these grounds, I would recommend subcutaneous enoxaparin (Option D) for DVT prophylaxis in this patient, recognizing it is important to carefully observe this patient for signs of potential bleeding or other side effects of anticoagulation. These risks need to be managed actively in the context of the patient's overall health status and the high risk of postoperative DVT.

**AI_D:** RESTATING THE QUESTION: A 72-year-old man who recently underwent knee replacement surgery is being observed in an intensive care unit. He has a medical history of coronary artery disease, impoverished heart performance (indicated by ejection fraction of 0.2), and is currently medicated with cardiovascular drugs and analgesic pump. His vital signs are stable. The question we are to answer is to determine the most beneficial prophylactic treatment for deep venous thrombosis (DVT), considering five potential options: the application of pneumatic compression devices, intravenous heparin, oral warfarin, subcutaneous enoxaparin, and subcutaneous heparin. DVT, often the result of surgery, refers to the formation of a blood clot in a deep vein, usually in the legs which can potentially be fatal if transferred to the lungs (pulmonary thromboembolism). PLANNING: We assume this patient with a compromised cardiovascular system and a history of coronary artery disease undergoing knee surgery is at a higher risk for DVT postoperatively. However, the use of anticoagulants in managing DVT could increase the risk of bleeding due to his poor heart performance indicated by the low ejection fraction (0.2). Let's consider an alternate assumption; effective DVT prevention in this postoperative period could help offset the risks associated with introducing anticoagulant therapy (heparin, warfarin, or enoxaparin) and using mechanical methods like pneumatic compression devices. Our primary objective should be to prevent DVT without further compromising the patient's cardiovascular health. CLINICAL REASONING: The delicate balance between mitigating the risk of bleeding and efficiently preventing DVT can drive our clinical reasoning. The patient's compromised cardiovascular health demands caution during the administration of anticoagulants, potentially elevating the risk of bleeding. However, postoperative patients are susceptible to bed rest and reduced mobility, which may potentially lead to DVT. Given the dire consequences of not preventing DVT and the dangerous implications of bleeding in our specific patient context, administering anticoagulants (eg: enoxaparin) in a controlled manner becomes a lesser evil. CONCLUDING: Embarking measures for the prevention of DVT are imperative in postoperative knee replacement patients, particularly in patients having a documented background of a compromised cardiovascular condition. While introducing anticoagulants can potentially increase the risk of bleeding, the benefits of preventing a potentially lethal complication (like pulmonary thromboembolism secondary to DVT) outweigh the risk, especially with careful clinical monitoring and dosage control. Therefore, a subcutaneous injection of enoxaparin (Option D) should be the most beneficial approach in this patient. RESPONSE: Reflecting on the clinical setting, I conclude that prophylactic treatment with subcutaneous enoxaparin (Option D) would likely be the most beneficial for this patient in preventing deep venous thrombosis. Despite the risk of bleeding associated with anticoagulants, careful dosage control and monitoring can reduce this risk while effectively preventing a potentially serious complication like DVT.

**Facilitator** **[Round 7]:** Summary: AI_A, AI_C, and AI_E all recommend the use of bilateral lower extremity pneumatic compression devices (Option A) for DVT prophylaxis in the patient. They argue that this non-pharmacological approach is safer for the patient given his history of coronary artery disease and low ejection fraction, as it avoids the bleeding risks associated with anticoagulant therapies. On the other hand, AI_B and AI_D suggest the use of subcutaneous enoxaparin (Option D) for DVT prophylaxis. They argue that despite the increased risk of bleeding, the efficacy of this pharmacological method in preventing DVT outweighs the risks, especially when the patient is closely monitored in the ICU. Key Differences: The key difference in the council members' perspectives lies in their prioritization of safety versus efficacy. AI_A, AI_C, and AI_E prioritize safety, choosing a method that minimizes the risk of bleeding due to the patient's cardiovascular issues. In contrast, AI_B and AI_D prioritize efficacy, arguing that the risk of DVT is so high in this patient that a more effective prophylaxis method is warranted, even if it carries a higher risk of bleeding. Question for Council Members: How do you weigh the importance of safety versus efficacy in this specific case, and how might your recommendation change if the patient's cardiovascular health were to improve or deteriorate?. Based on your preceding response, how would you answer the original question: [A 72-year-old man is admitted to the intensive care unit for pain management and observation of ventricular ectopy 4 hours after undergoing uncomplicated left total knee replacement. He is receiving fentanyl via systemic patient-controlled analgesic pump for pain management. Medical history is remarkable for coronary artery disease, for which he takes atorvastatin, furosemide, carvedilol, lisinopril, and 81-mg aspirin. Baseline echocardiogram showed an ejection fraction of 0.20. Vital signs now are temperature 38.0°C (100.4°F), pulse 90/min, respirations 16/min, and blood pressure 130/90 mm Hg. Pulse oximetry on room air shows an oxygen saturation of 96%. Physical examination discloses no unexpected abnormalities. Which of the following regimens for prophylaxis of deep venous thrombosis will likely be most beneficial in this patient? (A) Continuous application of bilateral lower extremity pneumatic compression devices (B) Continuous intravenous infusion of heparin titrated to a PTT of 1.5 to 2.0 times the control value (C) Oral warfarin (D) Subcutaneous enoxaparin (E) Subcutaneous heparin]

**AI_C:** RESTATING THE QUESTION: The query requests the most beneficial regimen to prevent deep venous thrombosis (DVT) in a 72-year-old male patient who has just undergone a knee replacement surgery. Five possible options are given, including (A) bilateral lower extremity pneumatic compression devices, (B) continuous intravenous infusion of heparin, (C) oral warfarin, (D) subcutaneous enoxaparin, and (E) subcutaneous heparin. The patient has a medical background notable for coronary artery disease and is exhibiting a low ejection fraction. PLANNING: Key assumptions include the significant risks of DVT following major orthopedic surgery and a possible heightened risk of bleeding due to anticoagulant therapy in the setting of the patient's coronary artery disease. Considering the patient's cardiovascular health, the decision between non-pharmacological and pharmacological approaches is key. The potential benefits of each option should be weighed against their respective risks and the patient's unique medical profile. CLINICAL REASONING: Total knee replacement surgery places the patient at an increased risk of DVT. Therefore, prophylaxis is crucial. However, the choice of prophylactic method should consider the patient's coronary artery disease and reduced ejection fraction. The use of anticoagulants could increase the risk of bleeding, potentially exacerbating the patient's cardiac condition. In contrast, mechanical prophylaxis, such as pneumatic compression devices, avoids the bleeding risk but might not be as effective. CONCLUDING: Given the circumstances, a balanced approach that maximizes DVT prophylaxis efficacy while minimizing risk to the patient is necessary. While anticoagulant therapy is generally effective for DVT prophylaxis, the patient's cardiac history may make mechanical prophylaxis a safer option, particularly in the immediate postoperative period. RESPONSE: Therefore, my recommendation is Option A: Continuous application of bilateral lower extremity pneumatic compression devices. This choice takes into account the specific medical history and current health status of the patient and balances the risk-benefit equation considering this patient's unique situation. Future decisions regarding anticoagulant therapy for DVT prophylaxis should be based on his cardiovascular function's improvement or deterioration.

**AI_A:** RESTATING THE QUESTION: The patient is a 72-year-old man who has just undergone a knee replacement surgery. In his medical history, he has coronary artery disease and a low ejection fraction, suggesting heart failure or previous heart injury. Post-operatively, he needs a strategy to prevent blood clots in the deep veins of his leg, known as deep vein thrombosis (DVT), which can lead to a life-threatening condition called pulmonary embolism (PE). Several strategies, including mechanical and pharmaceutical ones, are considered. PLANNING: Given his low ejection fraction, strategies that might increase his bleeding risk, particularly intravenous heparin and oral warfarin, need to be carefully considered. At the same time, he is recovering from surgery and has been immobilized, so there is an increased risk of DVT given his recent knee replacement. CLINICAL REASONING: The balance between preventing deep vein thrombosis and not increasing the risk of bleeding is delicate in this patient due to his coronary artery disease and knee surgery. From a safety perspective, the continuous application of bilateral lower extremity pneumatic compression devices (option A) reduces the risk of DVT without raising the risk of bleeding, making it the safest option. It also does not interact with his current medication. However, from an efficacy angle, Subcutaneous enoxaparin (option D) is an effective anticoagulant that greatly reduces the risk of DVT and would be beneficial if his risk for DVT is determined to be significantly high. CONCLUDING: The decision should be based on the individual patient's risk factors for both DVT and bleeding. Given the patient's heart condition, prioritizing safety seems to be reasonable. However, if his cardiovascular status improves significantly, the more effective anticoagulant option could be taken into consideration. RESPONSE: Therefore, the best course for DVT prophylaxis for this specific patient, at this point in his recovery, is likely the Continuous application of bilateral lower extremity pneumatic compression devices (option A) given his overall clinical picture and current cardiovascular status. However, it should be emphasized that the appropriateness of DVT prevention strategies relies heavily on a careful, patient-specific risks and benefits analysis.

**AI_D:** The question at hand concerns the optimal approach to prevent deep venous thrombosis (DVT) in a 72-year-old man who has recently undergone knee replacement surgery and has a significant cardiac comorbidity. Several prophylactic options have been proposed, including mechanical methods such as bilateral lower extremity pneumatic compression devices (Option A), and pharmacological interventions such as intravenous heparin (Option B), oral warfarin (Option C), subcutaneous enoxaparin (Option D), and subcutaneous heparin (Option E). When planning the preventive strategy, it is important to consider this patient's individual characteristics. The patient has a history of coronary artery disease and a low ejection fraction, which increase his risk for both venous thromboembolism and bleeding. This necessitates a careful balance between these risks when choosing the appropriate prophylaxis regimen. Though pharmacological methods of DVT prophylaxis, particularly injectable low molecular weight heparins like enoxaparin (Option D), are generally more effective, they also carry a higher risk of bleeding. These agents inhibit clot formation, which can lead to an increased risk of hemorrhage, potentially complicated by the concurrent use of aspirin in this patient. On the other hand, mechanical methods like pneumatic compression devices (Option A) don't carry a risk of bleeding and can be quite effective when used properly. These devices work by mechanically promoting venous return, thereby preventing stasis and reducing the risk of clot formation. This may be a safer alternative in a patient with a higher risk of bleeding. Connecting these concepts, it's worth noting that recent guidelines and studies advocate for the use of a pharmacological approach in orthopedic post-operative patients, even with elevated risk of bleeding, due to the high thrombotic risk associated with these procedures. However, given the significant cardiac comorbidity and baseline use of aspirin in this patient, I lean towards a more cautious approach. Therefore, my final response is to recommend the continuous application of bilateral lower extremity pneumatic compression devices (Option A) for this patient. This approach provides a safer alternative due to its minimal bleeding risk, while still addressing the patient's increased risk of DVT following surgery. However, the final decision should be individualized, incorporating the patient's values and preferences, as well as the evolving clinical scenario.

**AI_E:** 1) RESTATING THE QUESTION: The question asks about the most beneficial prophylaxis approach against deep venous thrombosis (DVT) after a knee replacement for a 72-year-old male patient. Several specific factors about his health status to consider are his use of a patient-controlled analgesic pump with fentanyl, his notable medical history including coronary artery disease and a low ejection fraction, and his medication list (atorvastatin, furosemide, carvedilol, lisinopril, and aspirin). Deep Vein Thrombosis (DVT) is the formation of a blood clot in a deep vein, often in the leg. 2) PLANNING: Two potential assumptions are that the patient's low ejection fraction and coronary artery disease necessitate a more careful approach for DVT prophylaxis, and that the patient's status, being in ICU, allows for more intensive management options. The first assumption could be questioned if we prioritize failure to prevent DVT over bleeding risk from pharmacological prophylaxis methods. Alternatively, we could question the second assumption if we prioritize less invasive methodologies given the patient's comorbidities. 3) CLINICAL REASONING: Our patient has a remarkable medical history of coronary artery disease and reduced ejection fraction. These are conditions associated with a higher risk of bleeding, especially when combined with anticoagulant therapy. Furthermore, his recent surgical intervention and analgesic pump indicate a need for optimal pain management during post-operative care, when the risk of DVT is elevated due to immobilization. However, the risk of bleeding associated with anticoagulants may outweigh their advantage as a preventive measure against DVT. This implies that we should balance between the risk and benefit of pharmacological and mechanical prophylaxis in this case. 4) CONCLUDING: In light of the patient's considerable cardiovascular risk factors, the most beneficial DVT prophylaxis would likely be a method that provides sufficient efficacy without compounding this risk. This method should ideally also offer comfort and allow for optimal pain management, as these factors may influence compliance and recovery. 5) RESPONSE: Thus, in this case, the likely most beneficial regimen for DVT prophylaxis is the continuous application of bilateral lower extremity pneumatic compression devices (Option A). This is because it effectively reduces DVT risk, without significantly increasing the risk for bleeding, which could be a serious complication given the patient's cardiovascular condition. This method is also non-invasive, allowing for better easier management and improved patient comfort.

**AI_B:** Restating the Question: The scenario involves a 72-year-old man who has recently undergone left total knee replacement and is at risk of developing deep vein thrombosis (DVT). He also has a history of heart disease and an impaired ejection fraction. We take into consideration multiple factors, including his medical history and current condition, to determine the best DVT prophylaxis regimen for him. The options range from non-pharmacological (pneumatic compression devices) to pharmacological measures (heparin, warfarin, enoxaparin). Planning: In choosing the most beneficial prophylaxis regimen for DVT in this case, we should consider the patient’s medical condition as well as the efficacy and safety profile of each choice. The risk of bleeding complications is a critical consideration due to his usage of anticoagulants for coronary heart disease and his reduced ejection fraction. As he is under intensive care, optimal management could consist of aggressive interventions, subject to close monitoring. Clinical Reasoning: Patients post knee arthroplasty are at a high risk of DVT, thus DVT prophylaxis is crucial. Pneumatic compression devices (Option A) are non-pharmacologic interventions and have shown to be effective in reducing the incidence of DVT with negligible risk for bleeding complications. However, their efficacy might be lower than other medical interventions. Heparin, warfarin, and enoxaparin (Options B, C, D, E) are pharmacological interventions with proven efficacy, but they come with an increased risk of bleeding, especially in patients with a history of coronary heart disease. Concluding: Given this patient's age, low ejection fraction, and use of anticoagulants, a non-pharmacological approach could be beneficial due to a lower risk of causing bleeding complications. However, medical interventions might be more effective in preventing DVT. Response: Considering the overall clinical scenario, the most beneficial prophylaxis for this patient would likely be (A) Continuous application of bilateral lower extremity pneumatic compression devices. Despite the high risk of DVT post knee arthroplasty, the patient’s other medical conditions, particularly coronary artery disease and a low ejection fraction, tilt the balance of benefits and risks towards minimizing the possibility of bleeding complications. This approach would also be consistent with the principles of providing patient-centered and risk-tailored prophylaxis.

**Facilitator** The consensus among the AI responses is that the most beneficial regimen for prophylaxis of deep venous thrombosis (DVT) in this 72-year-old patient, who has recently undergone knee replacement surgery and has a history of coronary artery disease with a low ejection fraction, would likely be the continuous application of bilateral lower extremity pneumatic compression devices (Option A). This recommendation is based on the patient's specific medical history and current health status, which necessitate a careful balance between the efficacy of DVT prophylaxis and the risk of bleeding complications. The pneumatic compression devices are a non-pharmacological intervention that reduces the risk of DVT without increasing the risk of bleeding, making it a safer option given the patient's cardiovascular condition. However, it's important to note that while this method is effective, it may not be as potent as pharmacological interventions such as heparin or enoxaparin. The decision to use this method should be based on a careful analysis of the patient's individual risk factors for both DVT and bleeding. If the patient's cardiovascular status improves significantly, a more effective anticoagulant option could be considered. However, given the current circumstances, prioritizing safety seems to be the most reasonable approach. It's also important to emphasize that the appropriateness of DVT prevention strategies relies heavily on a patient-specific risk and benefits analysis. The final decision should be individualized, incorporating the patient's values and preferences, as well as the evolving clinical scenario.
